# Controlled Release of Doxorubicin from the Drug Delivery Formulation Composed of Single-Walled Carbon Nanotubes and Congo Red: A Molecular Dynamics Study and Dynamic Light Scattering Analysis

**DOI:** 10.3390/pharmaceutics12070622

**Published:** 2020-07-03

**Authors:** Anna Jagusiak, Katarzyna Chlopas, Grzegorz Zemanek, Pawel Wolski, Tomasz Panczyk

**Affiliations:** 1 Faculty of Medicine, Chair of Medical Biochemistry, Jagiellonian University Medical College, Kopernika 7, 31-034 Krakow, Poland; katarzyna.chlopas@uj.edu.pl (K.C.); grzegorz.zemanek@uj.edu.pl (G.Z.); 2 Institute of Catalysis and Surface Chemistry, Polish Academy of Science, Niezapominajek 8, 30-239 Krakow, Poland; pawl.wolski@gmail.com (P.W.); panczyk@vega.umcs.lublin.pl (T.P.)

**Keywords:** self-assembled ribbon-like structures (SRLS), Congo red (CR), doxorubicin (DOX), single walled carboxylic acid functionalized carbon nanotubes (SWNT), dynamic light scattering (DLS), molecular dynamic (MD), radial distribution function (RDF)

## Abstract

The controlled delivery and release of drug molecules at specific targets increases the therapeutic efficacy of treatment. This paper presents a triple complex which is a new potential drug delivery system. Triple complex contains single-walled carbon nanotubes, Congo red, and doxorubicin. Nanotubes are built of a folded graphene layer providing a large surface for binding Congo red via “face-to-face” stacking which markedly increases the binding capacity of the carrier. Congo red is a compound that self-associates to form supramolecular ribbon-like structures, which are able to bind some drugs by intercalation. The nanotube–Congo red complex can bind the model drug doxorubicin. Thus, a new triple carrier system was obtained. The aim of this paper is to present studies on the controlled release of a model anticancer drug from a triple carrier system through pH changes. The specific aim of the study was to model the structure of the obtained experimental systems and to compare the changes in the average energy of interaction between its components induced by pH changes. The studies also aimed to compare the intensity of pH-dependent changes in hydrodynamic diameters of individual components of the triple carrier system. The effect of pH changes on the stability of the analyzed systems was examined using the molecular modeling method and dynamic light scattering. The decrease in pH influenced the structure and stability of the analyzed triple systems and ensured efficient drug release. The changes in hydrodynamic diameters of the obtained fractions were examined with the use of dynamic light scattering and were confirmed by computer simulation methods. The formulation presented in this paper shows potential for a therapeutic application owing to its high drug binding capacity and pH-dependent release. This ensures prolonged local action of the drug. The results reveal that the studied complex fulfills the basic requirements for its potential use as drug carrier, thus reducing side effects and enhancing pharmacological efficacy of drugs.

## 1. Introduction

Formulations that can be used for the targeted delivery and controlled release of drugs at predefined sites are currently a vital topic of research. The efficient release of the drug from the carrier presents a special challenge. Different drug release methods, like temperature, ionic strength, infrared radiation, or magnetic field have been tested [1,2,3,4,5,6,7]. One method described in the literature as useful for drug release is pH reduction. The drug remains bound to the carrier at pH = 7.4 and is released at the place of destination at lower pH, like in cancer tissues or after endocytosis of the delivery system [8,9,10,11].

**Figure 1:**
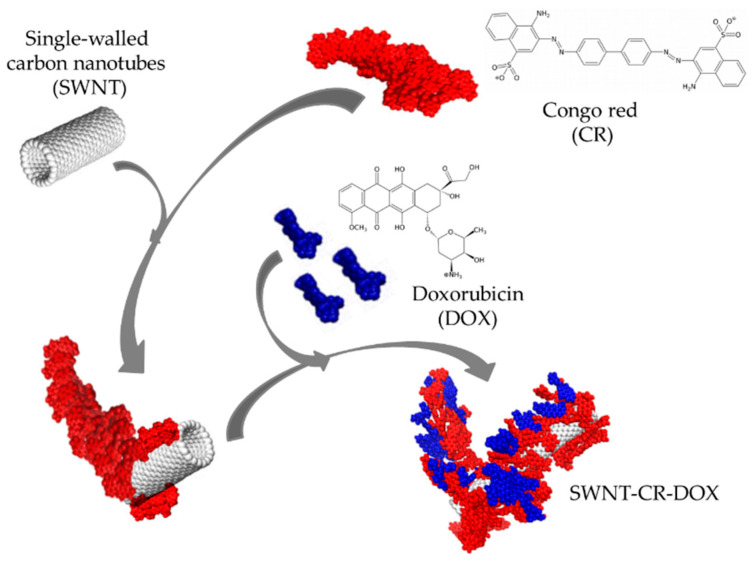
Schematic model of Congo red (CR) interaction with single-walled carbon nanotubes (SWNT) and intercalation of guest compound (doxorubicin, DOX) into ribbon-like structure of Congo red (CR).

A triple system composed of single-walled carbon nanotubes (SWNT), self-assembled ribbon-like structures (SRLS) of Congo red type (CR), and model drug doxorubicin (DOX) bound to them (Figure 1) is an example of a carrier capable of drug release under lower pH [12].

Some organic molecules are capable of self-association via non-covalent interactions. Such structures can form mono-, di-, or multimolecular associations. This phenomenon can also be observed within the structure of biological membranes and microtubules. Polyaromatic molecules of an elongated shape and bearing appropriately located polar groups interact to form elongated supramolecular ribbon-like systems which are referred to as self-assembled ribbon-like structures—SRLS. They are a special type of self-assembled systems [13]. SRLS composed of self-assembled diazo dyes of the CR type were studied as the systems are able to interact with proteins with a partly or totally destabilized structure [14,15]. Partial destabilization occurs in native proteins and is related to the function the protein performs. For instance, it can be observed in antibodies after antigen binding [16] or in complement system proteins, e.g., C1q after their binding to antibodies complexed with antigen [17] or in enzymes after ligand binding [13,18]. CR was used only as a model because its properties as SRLS are well known. In the future, it can be replaced by other, more biocompatible compounds with similar properties. An example of such a compound is Evans blue [19]. 

For some time, SRLS of the CR type have been tested for usability as carrier systems for targeted drug delivery. It is possible because SRLS are able to interact with some molecules with flat aromatic structure, including chemotherapeutics, like DOX [12,20]. Their ability to bind antibodies complexed with antigen paves the way for their application as carrier systems. 

Single-walled carbon nanotubes (SWNT) are described as structures with a potential application as drug carriers. The use of SWNT requires their initial dispersion because due to a strongly hydrophobic character and thread-like structure, they are not spontaneously dispersed in aqueous solutions. Thus, functionalization of the surface of carbon nanotubes is used for this purpose. The functionalization affects the outer surface of carbon nanotubes either by covalent or noncovalent interactions [21,22,23]. 

Ligands covalently bind to nanotubes at sites of the so-called structural defects (sites of covalent modifications) on the surface or edges of nanotubes [24,25,26].

Ligands can also interact with the nanotube surface via noncovalent interactions, which, in contrast to covalent binding, have the benefit of leaving the nanotube surface unmodified, thus preserving its structure and, e.g., electronic properties. These interactions include van der Waals interactions, π-π stacking, and hydrogen bonding [27]. Noncovalent interactions can also be engaged in the overbraiding of nanotubes by surfactants (anionic, cationic, and non-ionic) and larger molecules, such as polymers or biopolymers (e.g., nucleic acids) [28]. Sodium dodecyl sulfate (SDS) [29], synthetic peptides with a hydrophobic part (interacting with the nanotube surface) and hydrophilic part (increasing solubility in aqueous environment) [30], mixtures of cationic lipids [31], or cholesterol derivatives [32] are also used for the dispersion of carbon nanotubes. Compounds of cationic nature allow for additionally efficient DNA binding. The nanotubes were also functionalized by polysaccharides (sodium alginate and chitosan) [33]. 

The functionalization process is not only helpful for the dispersion of nanotubes in aqueous solutions but also for the binding of therapeutic molecules, ligands for targeted transport or fluorescent compounds used to follow the fate of nanotubes in the cell. These compounds can be covalently or noncovalently bound both on the whole nanotube surface and on its edges [25]. Contrary to spherical nanoparticles, the carbon nanotubes have a large inner space which can be filled with drugs [33], like cisplatin [34]. The nanotube ends can be closed by fullerenes C_60_ [35]. There are also reports about intercalation of other nanoparticles, peptides, or proteins in the nanotube interior. The advantage of entrapment of compounds inside the nanotubes is their protection in the nanotube interior and possibility of controlled release [36]. 

Therefore, nanotubes are an attractive material that can be applied for therapeutic purposes, capable of binding peptides, proteins, nucleic acids, and different drugs. 

It was documented that Congo red could be bound on the nanotube surface and an attempt was made to explain the mechanism of this interaction. Molecular modeling studies indicated face-to-face binding of supramolecular ribbon-like CR structures to the nanotube surface. It indicates that CR molecules bind directly to the nanotube surface and then other CR molecules continue to attach to them thus creating protruding supramolecular structures. This allows for the binding of considerable amounts of CR to the nanotube surface which significantly increases capacity of drug binding to the carrier.

The results of experimental studies documenting CR binding to SWNT were presented in the paper by Jagusiak et al. [37], while molecular modeling of these systems was described in the article by Panczyk et al. [38]. The triple SWNT-CR-DOX systems created after binding of the model drug doxorubicin to the carbon nanotube–Congo red complex were characterized in the paper by Jagusiak et al. [12], though without describing these interactions with the use of the molecular modeling tool.

Available literature contains some information about molecular dynamics simulation studies of both Congo red and doxorubicin in aqueous solutions [39]. Recently, the results of studies on the interactions between different supramolecular systems (Congo red, Evans blue, Titan yellow) and doxorubicin have been presented [20]. In addition, modeling of DOX interaction with some selected dyes (e.g., methyl red, neutral red, p-phenylenediamine and bromothymol blue) and carbon nanotubes has been described along with evaluation of the effect of lowered pH on drug release from such systems. The release of neutral red-DOX complex or free DOX from carbon nanotube surface was observed to occur after pH reduction [40]. However, to the best of our knowledge, there are no papers describing molecular dynamics study of the interactions between SRLS creating characteristic ribbon-like structures, taking CR as an example since it forms unique CR-DOX complexes with carbon nanotubes. Hence, the presentation of such studies will broaden the knowledge of such interactions and will allow for the verification of hypotheses on drug release at reduced pH based on experimental data.

Previous analysis of the data from molecular modeling studies confirmed that ring-to-ring interaction in a face-to-face fashion was an optimal manner of CR interaction with the SWNT surface. The conclusions from investigations into the properties of SWNT-CR system suggested significant pH-dependent changes in the structure of adsorbed CR [38]. Therefore, it seems feasible that pH-induced changes can be used as a factor controlling the structure and range of interactions of different molecules with the SWNT-CR system. To verify this hypothesis, a triple SWNT-CR-DOX system was analyzed at neutral pH and at pH lowered to the value observed in the tumor tissue, with the use of dynamic light scattering (DLS). Hydrodynamic diameters of the SWNT-CR-DOX system and its components under different pH conditions were compared. Calculations were also conducted to examine the structure and properties of these systems by analysis of mean energies of interaction between different components of the system at various pH conditions. The results obtained by computer simulation methods allow for a deeper understanding of the impact of pH of the milieu on the microscopic structure and stability of the studied systems.

## 2. Materials and Methods 

### 2.1. Materials

Congo Red (CR, 96% purity, Aldrich Chemical Company, Inc. Milwaukee, WI, USA), single walled carbon nanotubes, carboxylic acid functionalized (SWNT, purity > 90%, length 0.5–1.5 µm, diameter 4–5 nm, Sigma-Aldrich, Co., 3050 Spruce Street, St. Louis, MO, USA), and doxorubicin hydrochloride (DOX, Sigma-Aldrich, Co., 3050 Spruce Street, St. Louis, MO, USA) were used. All other reagents used were of analytical grade and were purchased from commercial sources.

### 2.2. Methods

#### 2.2.1. Preparation of SWNT-CR and SWNT-CR-DOX Complexes for DLS Analysis

##### Formation of SWNT-CR Complexes

SWNT (1mg/mL) in PBS with 0.9% NaCl, pH = 7.4 were sonicated (in a cool water bath) for 1 h. CR (5 mM) in PBS with 0.9% NaCl, pH = 7.4 was heated to 100 °C for 2 min, and then slowly cooled to a room temperature for 10 min. CR prepared in this way was combined with SWNT at 1:1 ratio by adding 1mL CR to 1 mL SWNT. Subsequently, the mixture was sonicated (in a cool water bath) for 1 h. After this time, the mixture was left in room temperature, without sonication, for 24 h to stabilize. The samples prepared in this way were divided into two equal parts. The first part was twice diluted with PBS buffer with 0.9% NaCl (pH = 7.4) to keep the sample pH neutral. The second part was twice diluted by addition of PBS with 0.9% NaCl (pH = 7.4) mixed at adequate proportion with 0.05 M acetate buffer (pH = 4.0) with 0.9% NaCl to obtain a final pH = 5.3. The SWNT-CR complex was separated from the unbound CR by filtration on Omnipore PTFE membrane filter (0.2 µm pore size; Merck Millipore, Darmstadt, Germany from the known amount of CR added and by measuring absorbance of free CR in the filtrate at 489 nm (extinction coefficient, ε_489_ = 50.46 cm^−1^ mM^−1^). Because nanotubes with carboxyl groups were used, which are easily dispersed in aqueous solution, 100% binding of added CR was obtained.

##### Formation of SWNT-CR-DOX Complexes

DOX (1 mM) in PBS buffer, pH = 7.4 was added to the SWNT-CR solution prepared according to the above procedure, thus, SWNT-CR-DOX complex was obtained. The SWNT-CR-DOX complex was separated from the unbound DOX by filtration on AmiconUltra filtration tubes (MWCO 50 kDa, Merck Millipore Ltd., Tullagreen, Carrigtwohill Co., Cork, Ireland) according to the procedure described in [12]. The amount of DOX bound was calculated based on measuring fluorescence of free DOX in the filtrate (Ex = 470 nm, Em = 550 nm) and reading the result from a calibration curve [12]. Because the previously optimized CR:DOX (2.5:1) ratio was used, at which DOX is completely bound to CR, 100% DOX binding to the triple complex was achieved. The SWNT-CR-DOX samples prepared in this way, after filtration and assessment that the total amount of CR and DOX added at pH 7.4 were bound, were divided into two equal parts. The first part was diluted 1:1 with PBS buffer (pH 7.4) to keep the sample pH neutral. The second part was diluted 1:1 with buffer with a correspondingly lower pH to obtain a final pH = 5.3. 

#### 2.2.2. Dynamic Light Scattering (DLS) 

Hydrodynamic radii were measured with the use of a dynamic light scattering (DLS) detector Zetasizer Nano ZSP (Malvern, Malvern, UK) with laser incident beam at λ = 633 nm and a fixed scattering angle of 173°. The measurement of each sample was performed at a temperature of 25 °C after 3-min incubation in the DLS instrument. A measurement comprised 9 repetitions each of which was an average of 10 records measured for 9 sec. Outliers were rejected from analysis and the results were averaged.

Continuous measurement of the hydrodynamic radius of SWNT-CR-DOX sample was conducted during pH change from 7.4 to 4.0 at the interval of 0.2 pH value. A titrator device MPT-2 was applied with three independent containers for titrating solutions (0.25 M HCl for initial acidification and 0.05 M HCl for precise pH adjustment and 0.25 M NaOH) and with a degassing unit for greater efficiency and precision of titration. The SWNT-CR-DOX sample, with the initial pH = 7.4, was prepared as described in Section 2.2.1, but additionally diluted 12 times. The solution to be titrated (12 mL) was placed in a container and hydrodynamic diameter was measured based on the values of intensity and number using a flow-through cell.

#### 2.2.3. Scanning Electron Microscopy (SEM)

Scanning electron microscopy was conducted on Jeol JSM-7500F, JEOL USA, Peabody, MA, USA. The samples in the form of 1 µL droplet were placed onto a 300-mesh copper grid and dried overnight in a desiccator.

#### 2.2.4. Molecular Dynamics (MD)

In the present paper, molecular dynamics (MD) method was used for characterization of the studied systems. Literature reports describe two approaches to parametrization of potential energy function (the so-called force field) of Congo red, i.e., CHARMM (chemistry at Harvard macromolecular mechanics) [41] and AMBER (assisted model building with energy refinement) [42] force fields. In the present calculations, general AMBER force field, GAFF (generalized AMBER force field) [43] was used due to its compatibility with other components of the systems under study, i.e., doxorubicin and carbon nanotubes. The force field parameters were compared and partially adopted from papers of other authors published earlier. Subsequently they were verified using antechamber program [44] which on the basis of molecular topology creates initial AMBER force field for a given molecule. Restrained electrostatic potentials (RESP) [45] for Congo red were calculated according to the same procedure as in [46] and the same approach was used for doxorubicin. Thus, both sets of partial charges were calculated using the RESP ESP charge derive procedure [47]. The obtained force field parameters for doxorubicin and Congo red are also available in ref. [20].

The calculations were carried out assuming three pH values of solution. These were formally called neutral, medium, and acidic. This is because it is impossible to set a strict value of pH in such type of computations. Thus, the neutral pH means that real pH is close to 7.4 and in such a case all CR molecules are in their unprotonated forms. The acidic pH means that all CR molecules are in the protonated form and it should experimentally correspond to pH lower than ca. 5.5. The medium value of pH corresponds to a situation when half of the CR molecules are in the unprotonated form and the second half in the protonated form. 

Electrostatic interactions were calculated using the Ewald summation method. Solvent molecules were included in the analysis explicitly using TIP3P water model [48]. Two types of SWNT were analyzed: narrow with chirality (10.0) and wide with chirality (30.0). Diameters of nanotubes were 7.8 Å and 23.5 Å, respectively, while their length was 82 Å. The force field associated with SWNT was based on the use of aiREBO potential [49]. The aiREBO potential belongs to the group of reactive potentials and is one of the most advanced potentials created for description of carbon materials. It allows for modeling of carbon nanotube as a flexible object and preserves cylindrical structure of nanotube without additional bonds. 

The calculations were conducted using the open source LAMMPS code [50] (Large-scale Atomic/Molecular Massively Parallel Simulator). One single-walled carbon nanotube with chirality (10.0) or (30.0) was placed in the simulation box. They comprised 800 or 2400 carbon atoms, respectively. Depending on the dimensions of the studied nanotube, 20, 40, or 50 CR molecules, 10 or 20 DOX molecules, and around 24,000 water molecules were placed in the simulation box. At the beginning of simulation, CR and DOX molecules were uniformly distributed inside the box and no molecules were placed inside the nanotube. During simulation water molecules were treated as rigid molecules using the SHAKE algorithm. The numbers of Cl^−^ and Na^+^ ions added to the simulation box were adequate to achieve the assumed ionic strength and pH value. Cutoff radius for van der Waals interactions and real component of electrostatic interactions was 12 Å.

The calculations were conducted using 1.5 fs timestep. The systems were kept at a constant temperature of 310 K and constant pressure of 1 bar using a Nose-Hoover barostat. The systems were equilibrated at a temperature of 400 K for 1.2 ns and next were cooled down to 310 K over 0.6 ns, followed by another equilibration stage lasting 0.9 ns. After this equilibration cycle production runs were carried out lasting typically 1.5 ns. 

Doxorubicin can occur in one of three forms: cationic, neutral, and anionic. In the presented calculations, the analyzed pH values did not exceed pH 7.4. Thus, in all studied systems, doxorubicin occurred as an ion with a resultant positive charge, with protonated amine group [51]. In every case, adequate amounts of Na^+^ and Cl^−^ ions were included in the simulation box in order to maintain the ionic strength of solution at the assumed level and to maintain the neutral charge of the system.

## 3. Results and Discussion

### 3.1. The Effect of pH on DOX Release from a Triple SWNT-CR-DOX Complex (DLS Analysis)

Two sample concentrations were used. The higher concentration was used for hydrodynamic diameter analysis by DLS at two pH points (pH 7.4 and pH 5.3). The lower concentration (12 times diluted sample in pH 7.4) was used for the continuous analysis of hydrodynamic diameters by the DLS method using a titrator for pH changes. At higher concentrations, smaller compounds (CR, DOX and CR-DOX) are visible. On the other hand, the lower concentration in the measurement of hydrodynamic diameter changes using the DLS method with a titrator allows for better observation of SWNT complexes (with CR and CR-DOX). 

**Figure 2:**
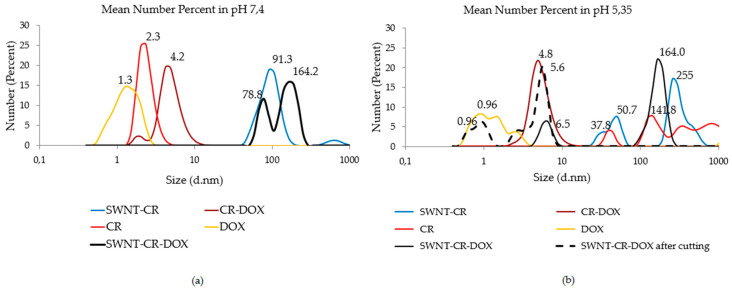
Hydrodynamic diameter distribution measurements (CR, DOX, CR-DOX, SWNT-CR, SWNT-CR-DOX and SWNT-CR-DOX after rejection of aggregates larger than 40 nm—dashed black line). Mean number percent at: (**a**) pH 7.4 (**b**) pH 5.35.

Two pH values were chosen to analyze drug release from the triple SWNT-CR-DOX system, namely pH = 7.4 corresponding to neutral pH and pH = 5.35 which is characteristic of cancer tissue. Samples were analyzed with the use of DLS method by measuring the changes in the hydrodynamic diameters of different components (CR, DOX, CR-DOX, SWNT-CR, and SWNT-CR-DOX) at both above-mentioned pH values (Figure 2). In the case of the SWNT-CR-DOX sample at reduced pH = 5.35, besides large aggregates (164 mm), additionally, one uniform peak was observed corresponding to the size of the CR-DOX complex (6.5 nm) [12]. In addition, analysis involved rejection of aggregates larger than 40 nm thus revealing peaks corresponding to smaller-sized particles, like CR-DOX (5.6 nm) and free DOX (0.96 nm). 

It was observed that free CR precipitated at the reduced pH. This effect was not observed for CR-DOX complex and free DOX.

**Figure 3:**
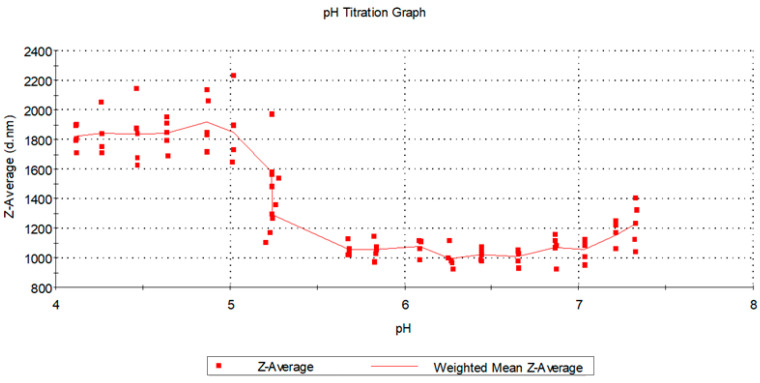
pH titration graph (in the range from 4.2–7.4) for SWNT-CR-DOX—Weighted Mean Intensity of Z-Average.

Furthermore, continuous measurement of the hydrodynamic diameter of the 12 times diluted sample by number upon titration was performed to analyze the system during gradually changing pH conditions. pH was reduced by 0.2 unit at a time and the obtained results were classified into three ranges: (1) pH 7.33–5.68; (2) pH 5.20–5.24; (3) pH 5.00–4.00, according to Figure 3. 

**Figure 4:**
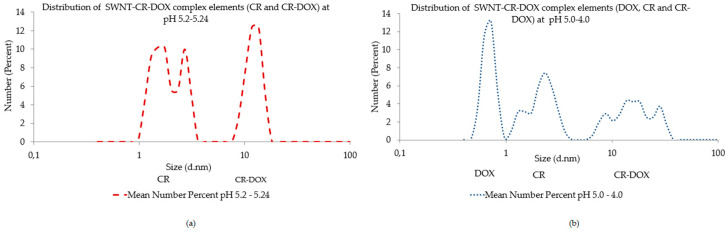
Hydrodynamic diameter distribution measurements. (**a**) number at pH 5.2–5.24 after rejection of large aggregates of more than 100 nm diameter, the release of CR-DOX complexes and free CR can be observed (the average of 5 measurements is presented) (**b**) number at pH 5.0–4.0 after rejection of large aggregates of more than nm diameter, the release of free DOX can be observed (the average of 12 measurements is presented).

When larger aggregates of more than 100 nm diameter, shielding smaller peaks, were rejected from analysis, the appearance of a peak has been observed, interpreted as the release of CR-DOX complexes at pH 5.2–5.24 (Figure 4a). As pH was reduced further in the range of pH 5.0–4.0, the appearance of a peak has been observed, interpreted as the release of the release of free DOX (Figure 4b).

The overall result of gradual pH reduction for the triple SWNT-CR-DOX complex showed a leap in the pH range from 5.8–5.0, connected with a tendency of the sample to aggregate in this pH range, which is presented in Figure 3. Before dilution of samples, smaller hydrodynamic diameters in the range of 0.1–200 nm were recorded. After sample dilution, only large hydrodynamic diameters (in the range 800–2000 nm) were recorded. The measurement device did not register peaks for small hydrodynamic diameter after dilution, as they were visible only after artificially cutting off peaks above 100 nm. The influence of sample dilution on the obtained particle size results is described in the literature [52]. 

### 3.2. Co-Adsorption of CR with DOX on SWNT (MD Analysis)

**Figure 5:**
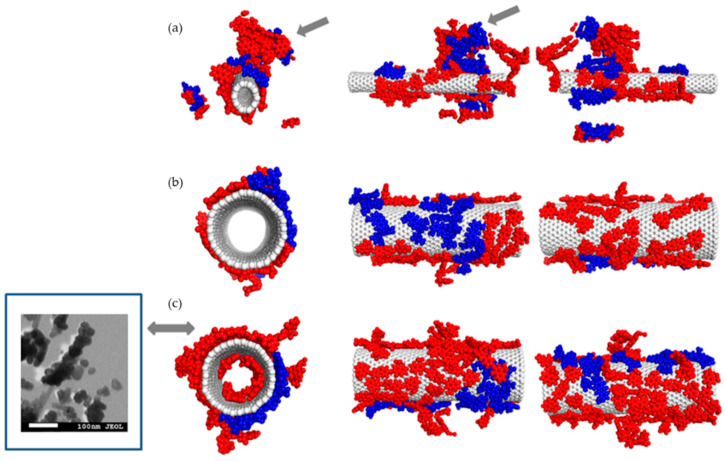
The structure of SWNT-CR-DOX systems at neutral pH values. (**a**) system composed of SWNT (10.0), 20 CR molecules (red) and 10 DOX molecules (blue), arrows show partial incorporation of DOX into supramolecular CR structure; (**b**) system composed of SWNT (30.0), 20 CR molecules and 10 DOX molecules; (**c**) system composed of SWNT (30.0), 40 CR molecules and 10 DOX molecules. Ionic strength of the solution was 0.145 M. Inset: scanning electron microscope photograph of nanotube (10 nm wide) with CR-DOX complex bound to the surface (concentration of CR = 1 mg/mL).

**Figure 6:**
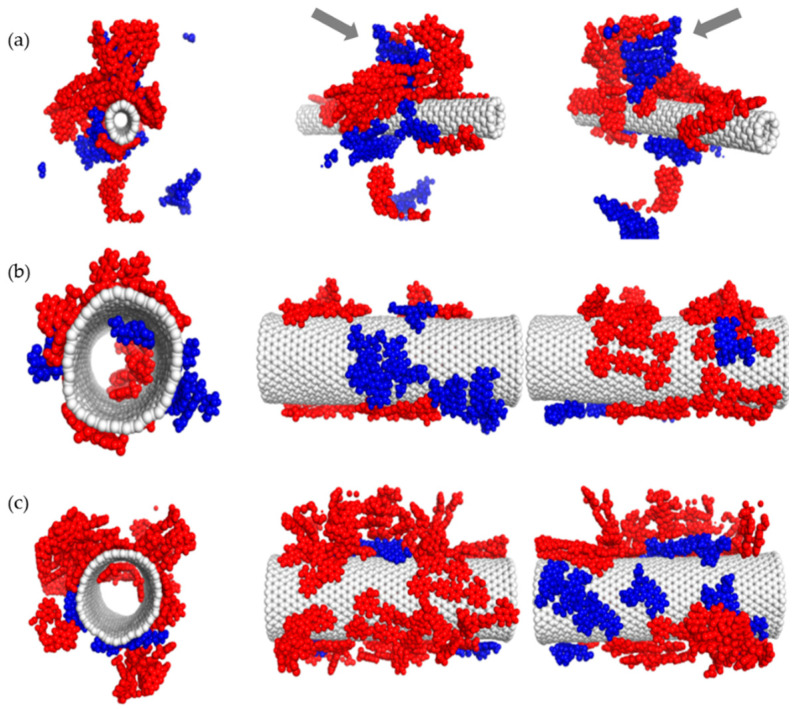
The structure of SWNT-CR-DOX systems at medium pH values (5.0 < pH < 7.4). (**a**) system composed of SWNT (10.0), 20 CR molecules (red) and 10 DOX molecules (blue), arrows show partial incorporation of DOX into supramolecular CR structure; (**b**) system composed of SWNT (30.0), 20 CR molecules and 10 DOX molecules; (**c**) system composed of SWNT (30.0), 40 CR molecules and 10 DOX molecules. Ionic strength of the solution was 0.145 M.

**Figure 7:**
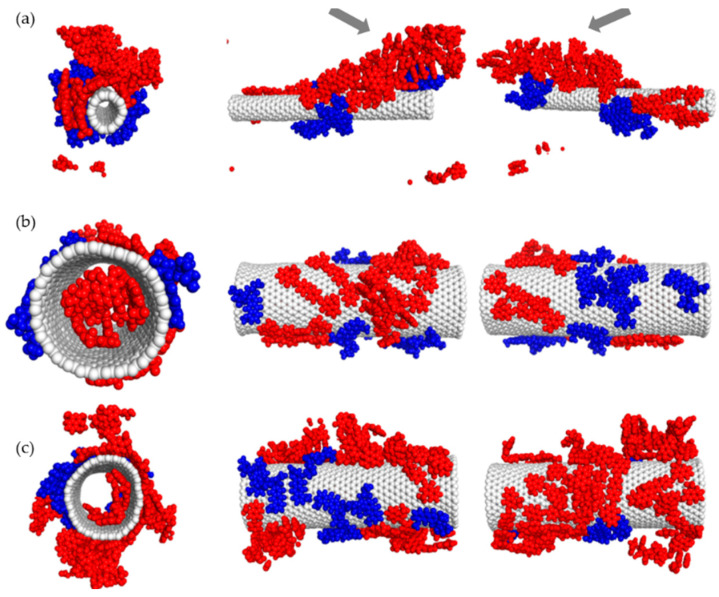
The structure of SWNT-CR-DOX systems at pH < 5.0. (**a**) system composed of SWNT (10.0), 20 CR molecules (red) and 10 DOX molecules (blue), arrows show ribbon-like CR structure; (**b**) system composed of SWNT (30.0), 20 CR molecules and 10 DOX molecules; (**c**) system composed of SWNT (30.0), 40 CR molecules and 10 DOX molecules. Ionic strength of the solution was 0.145 M.

**Figure 8:**
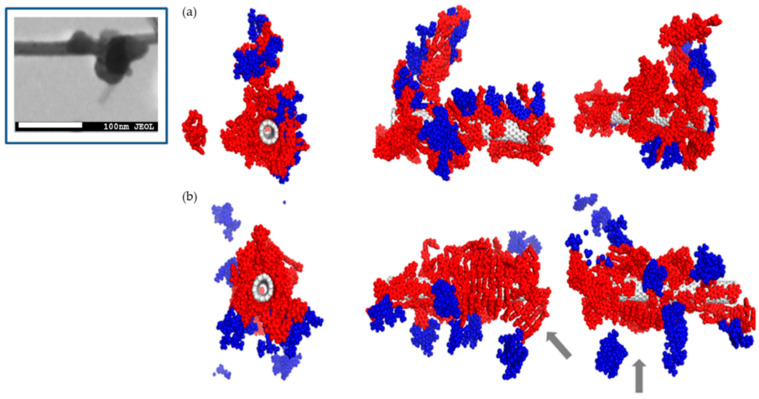
The structure of SWNT-CR-DOX systems at neutral pH (**a**) and acidic pH (**b**). The system contains narrow carbon nanotubes (10.0), 50 CR molecules (red) and 20 DOX molecules (blue). Ionic strength of the solutions is 0.264 M. Arrows show ribbon-like CR structure. Inset: scanning electron microscope photograph of nanotube (10 nm wide) with CR-DOX complex bound to the surface (higher concentration of CR = 5 mg/mL) at neutral pH.

For the purpose of verifying the experimental observations obtained with DLS method, a series of experiments was conducted with the use of molecular dynamics method. Figure 5, Figure 6, Figure 7 and Figure 8 illustrate geometry of the triple SWNT-CR-DOX system, i.e., they represent equilibrium states for defined temperature, ionic strength, and pH conditions. Top parts of Figure 5, Figure 6 and Figure 7 present systems built of a narrow carbon nanotube (10.0) and 20 CR molecules. Middle and bottom parts of these figures show nanotubes of a larger diameter (30.0), supplemented with 20 (middle part of the figures) and 40 (bottom part of the figures) CR molecules. To each of these systems, 10 DOX molecules were added. In addition, the inset in Figure 5 demonstrates a scanning electron microscope photograph of a wider nanotube (10 nm wide) with CR-DOX complex (with low concentration of CR = 1mg/mL) bound to its surface. Figure 8 presents the system composed of a narrow nanotube (10.0) containing larger amounts of CR (50 molecules) and DOX (20 molecules). In addition, the inset in Figure 8 demonstrates a scanning electron microscope photograph of a wider nanotube (10 nm wide) with CR-DOX complex (with high concentration of CR = 5 mg/mL) bound to its surface. 

Analysis of Figure 5, Figure 6 and Figure 7 leads to interesting conclusions about the structure of SWNT-CR-DOX complexes formed in these experiments. In the presence of narrow nanotube (10.0) Congo red partially preserves supramolecular structures at each tested pH value. At acidic pH (Figure 8), the ribbon-like arrangement of supramolecular structure created by CR molecules is clearly visible (indicated by arrows in the figure). It can also be seen that the ordering of the structures under study decreases as pH rises. At these conditions, DOX partially incorporates into the ribbon-like supramolecular CR structure and partially adsorbs directly on the nanotube surface. It can be clearly seen that DOX also forms aggregates both on the SWNT surface and within supramolecular CR ribbon.

When the wider nanotube (30.0) and a lower number of CR molecules (20 molecules) were used, both CR and DOX molecules were adsorbed individually on the nanotube surface and seemed to form two immiscible phases. The picture was slightly different when 40 CR molecules were present in the system. In this case, the structure of CR adsorbed on the surface clearly depended on pH, namely, as pH declined, CR molecules tended to group into three-dimensional aggregates. It was connected with limited available surface and increased energy of interaction between CR molecules. It appears that further increase in the number of CR molecules in the system would lead to formation of the structures similar to those observed for the narrow nanotube (10.0), i.e., to formation of the ribbon-like structure attached to the nanotube surface. Doxorubicin prefers to adsorb on the nanotube surface in a way maximizing its contact with the nanotube surface. In all cases, a clear segregation of CR and DOX was observed. Both components formed immiscible phases. It can also be noticed that, like in the case of earlier analyzed SWNT-CR complexes [38], nanotubes with chirality (30.0) undergo significant deformations. These deformations result from the interaction of nanotubes with CR, DOX and water molecules (for a better clarity, water molecules were not shown in the figures). However, this effect has no significant impact on the structure of the CR phase and DOX phase on their surface. Similarly, as before, the inner space of the narrow nanotubes (10.0) was not available to CR or DOX molecules. However, in the case of wider nanotubes (30.0), a certain number of CR and DOX molecules were observed in the nanotube interior.

Figure 8 illustrates the structures of systems formed in the presence of a greater density of CR and DOX. In this case, the system contained a narrow nanotube (10.0) and 50 CR molecules and 20 DOX molecules. The calculations were performed for neutral and acidic pH. Here elaborate supramolecular CR structures were observed and practically the entire nanotube surface was covered by CR and DOX molecules. A relationship between the ordering of the structures formed and pH value seems to be also preserved. In acidic pH, ribbon-like arrangement of CR molecules tightly adjacent to each other is evident while in neutral pH, CR molecules seem to form less ordered spatial forms.

pH change also distinctly influences behavior of DOX molecules. It can be seen that at higher pH values, doxorubicin molecules are located within extensive CR structures. pH reduction results in detachment of few-molecule-size DOX aggregates from CR-SWNT structures. 

Analysis of parameters collected in Table 1 confirms the conclusions based on visualization of the systems’ structures (Figure 5, Figure 6, Figure 7 and Figure 8). The effects related to DOX interaction with other components of the system are particularly interesting. Analysis of energy of DOX interaction with SWNT shows that the nanotube diameter is the most significant factor influencing this value. Nanotubes (30.0) provide for a direct DOX adsorption on their surface, thus much higher energies of 140~180 kJ mol^−1^ are observed. Nanotubes (10.0) have much smaller surface area. Therefore, much fewer DOX molecules can directly attach to the SWNT surface. In addition, they have to compete with CR for access to the surface, while CR in most cases shows higher adsorption energy than DOX. Only in the case of SWNT (10.0) + 20CR + 10DOX these energies are similar due to a tendency of CR to form supramolecular ribbon-like structures which exposes parts of the nanotube surface.

The most interesting effects are observed in relation to the pH-dependent changes in the energy of interaction of CR with DOX. At higher pH, these energies reach moderate values, which indicate that both molecules remain close to each other during simulation and are subject to attractive interactions of medium strength. pH lowering causes that this energy component practically disappears, thus, CR and DOX molecules move away from each other to a distance exceeding the range of interaction. This is a very interesting effect which was hereinafter subjected to detailed analysis based on radial distribution functions (RDF).

**Figure 9:**
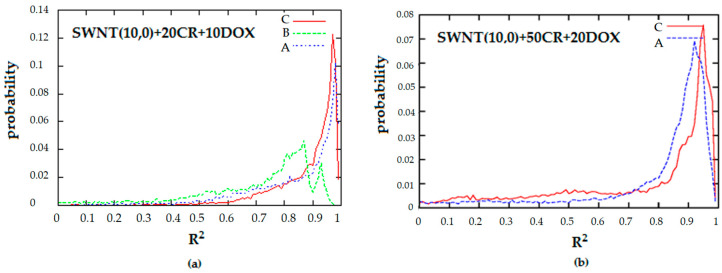
Histograms of the planarity of a single CR molecule, R^2^. (**a**) SWNT (10.0) complexed with 20 molecules of CR and 10 molecules of DOX; (**b**) SWNT (10.0) complexed with 50 molecules of CR and 20 molecules of DOX; (A—neutral pH, B—medium pH, C—acidic pH).

**Figure 10:**
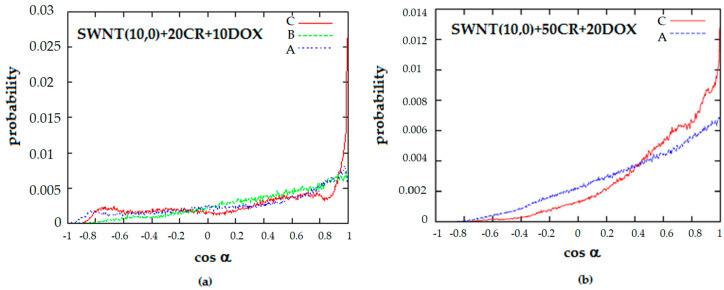
Histograms of cosine of the angle between two CR molecules; (**a**) SWNT (10.0) complexed with 20 molecules of CR and 10 molecules of DOX; (**b**) SWNT (10.0) complexed with 50 molecules of CR and 20 molecules of DOX; (A—neutral pH, B—medium pH, C—acidic pH).

The systems SWNT-CR-DOX containing nanotube (10.0) seem to form ordered ribbon-like structures though qualitative analysis of simulation snapshots alone may be biased. For this reason, histograms of *R^2^* (1) and cos *α* (3) were calculated for these systems which are presented in Figure 9 and Figure 10. These two parameters, which allow for quantitative description of the system structure, were used for the present analysis because a static picture based on snapshots does not provide information about long-term behavior of order parameters. 

The first parameter depicts the planarity of a CR molecule. It was defined as the coefficient of determination R^2^ obtained by fitting a plane to the centers of aromatic carbons in a CR molecule.

(1) $$R^{2}=\frac{\sum_{i=1}^{n}{\left(ax_{i}+by_{i}+c-\frac{1}{n}\sum z_{i}\right)}^{2}}{\sum_{i=1}^{n}{\left(z_{i}-\frac{1}{n}\sum z_{i}\right)}^{2}}$$

where *x_i_*, *y_i_*_,_ and *z_i_* are coordinates of aromatic carbons in a single CR molecule, *n* is the number of these atoms in a molecule, and *a*, *b* and *c* are parameters (2) of the best fit plane in 3D crossing these atoms, i.e., parameters satisfying to the equation *ax* + *by* + *c*z + *d* = 0. Parameters of this equation were calculated using 3D linear regression method, they read:

(2) $$\begin{array}{c} a=-\frac{\sum xy{\left(\sum y\right)}^{2}-n\sum xz\sum y^{2}+n\sum xy\sum yz-\sum x\sum y\sum yz-\sum xy\sum y\sum z+\sum x\sum y^{2}\sum z}{-n{\left(\sum xy\right)}^{2}+2\sum x\sum xy\sum y-\sum x^{2}{\left(\sum y\right)}^{2}-{\left(\sum x\right)}^{2}\sum y^{2}+n\sum x^{2}\sum y^{2}}\\ b=-\frac{-n\sum xy\sum xz+\sum x\sum xz\sum y-{\left(\sum x\right)}^{2}\sum yz+n\sum x^{2}\sum yz+\sum x\sum xy\sum z-{\left(\sum x\right)}^{2}\sum y\sum z}{n{\left(\sum xy\right)}^{2}-2\sum x\sum xy\sum y+\sum x^{2}{\left(\sum y\right)}^{2}+{\left(\sum x\right)}^{2}\sum y^{2}-n\sum x^{2}\sum y^{2}}\\ c=-\frac{-\sum xy\sum xz\sum y+\sum x\sum xz\sum y^{2}-\sum x\sum xy\sum yz+\sum x^{2}\sum y\sum yz+{\left(\sum xy\right)}^{2}\sum z-\sum x^{2}\sum y^{2}\sum z}{-n{\left(\sum xy\right)}^{2}+2\sum x\sum xy\sum y-\sum x^{2}{\left(\sum y\right)}^{2}-{\left(\sum x\right)}^{2}\sum y^{2}+n\sum x^{2}\sum y^{2}} \end{array}$$

where a given sum goes through all coordinates of aromatic carbons in the molecule. 

The second parameter was chosen as the average cosine of the angle between two CR molecules (cos α). Angles between CR molecules were defined as the angles between vectors normal to the planes fitted to the position of the centers of aromatic carbon atoms in a CR molecule, i.e.,

(3) $$\cos\left(\alpha \right)=\frac{a_{1}a_{2}+b_{1}b_{2}+1}{\sqrt[{\;}]{a_{1}^{2}+b_{1}^{2}+1}\sqrt[{\;}]{a_{2}^{2}+b_{2}^{2}+1}}.$$

Cosines of the angles were averaged for each combination of two CR molecules and for time. Based on the histogram of R^2^ we can conclude that a significant majority of CR molecules maintain nearly planar configuration which is indicated by maximum probability of observation for R^2^ > 0.9. A certain (small) fraction of CR molecules deviate from planar configuration which is indicated by non-zero probability of observation of small R^2^ values. The highest deviation from planarity is observed for the system SWNT(10.0) + 20CR + 10DOX at medium pH where maximum of probability of observation is located in the range 0.8 ≤ R^2^ ≤ 0.9. On the other hand, when the system has a greater density of CR and DOX, pH reduction results in slightly stronger preservation of planarity by CR molecules (Figure 9). 

Histograms of cos α confirm that 0 deg is the dominant angle between any 2 CR molecules. Thus, parallel orientation of molecules is the most probable and the supramolecular ribbon-like configuration is preserved. Like in the case of R^2^ parameter, not all CR molecules are parallel to each other. A small fraction of molecules presents a perpendicular or even antiparallel orientation. It can also be noted that, at acidic pH, CR molecules are most ordered in the structures they form. In systems containing high density of CR, histogram of cos α is much fuzzier than in systems with fewer CR molecules. This fact should be interpreted as formation of supramolecular ribbon-like structure with twisted spatial arrangement. In such case, CR molecules located far apart cease to be parallel to each other although they are parallel to their closest neighbors (Figure 10).

**Figure 11:**
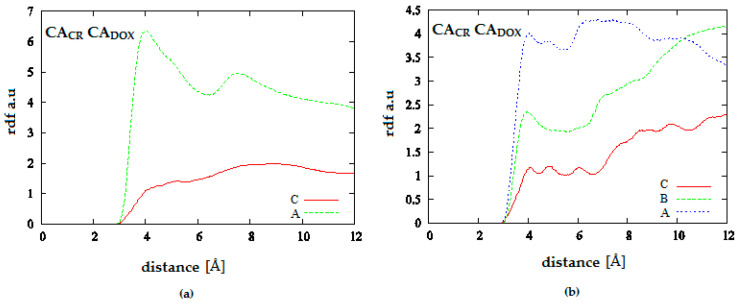
Radial distribution functions for the system: (**a**) SWNT (10.0) complexed with 20 molecules of CR and 10 molecules of DOX; (**b**) SWNT (10.0) complexed with 50 molecules of CR and 20 molecules of DOX; (A—neutral pH, B—medium pH, C—acidic pH).

Figure 11 presents radial distribution functions between CR and DOX molecules for systems containing carbon nanotube with chirality (10.0). These functions were defined as probability of observation of a given distance between any two aromatic carbons in both molecules (CA_CR_-CA_DOX_). Comparison of these distributions provides information about long-term distribution of densities of both molecules depending on the solution pH. Thus, in both cases, it can be observed that a higher pH facilitates close contact of both molecules. It can be assumed that DOX is incorporated in the spatial structure of CR. pH lowering causes a strong decrease in DOX density in close vicinity of CR. In the system with a large amount of CR, both components become considerably separated beyond the range of intermolecular interactions. 

**Figure 12:**
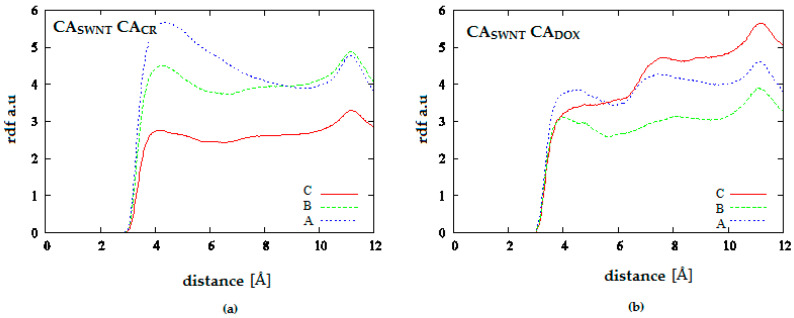
Radial distribution functions for the system SWNT (10.0) complexed with 20 molecules of CR and 10 molecules of DOX; (**a**) SWNT-CR interaction; (**b**) SWNT-DOX interaction; (A—neutral pH, B—medium pH, C—acidic pH).

**Figure 13:**
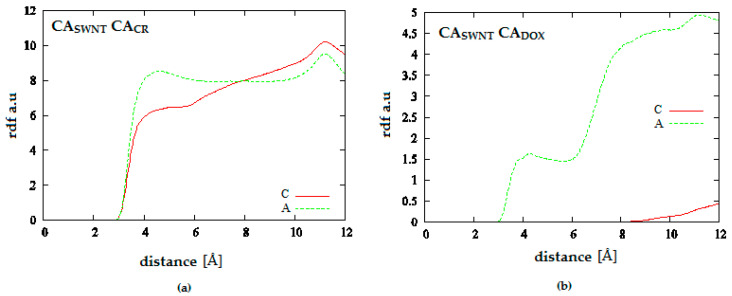
Radial distribution functions for the system SWNT (10.0) complexed with 50 molecules of CR and 20 molecules of DOX; (**a**) SWNT-CR interaction; (**b**) SWNT-DOX interaction; (A—neutral pH, C—acidic pH).

Figure 12 and Figure 13 provide additional information about the location of DOX molecules within the simulation box. They present analogical probability distributions but, in this case, for the distances between carbon atoms building nanotube and aromatic carbons in DOX molecule (CA_SWNT_-CA_DOX_) or CR molecule (CA_SWNT_-CA_CR_). Figure 12 shows the results for the system containing 20 CR molecules. It can be seen that CR molecules are located near the SWNT surface which corresponds to the maximum of the first peak. pH lowering results in decline of this maximum and increase in the second peak. The second peak corresponds to high density of CR molecules further away from the nanotube. Thus, it is the area where the supramolecular ribbon-like structure exists. DOX density is clearly lower in close vicinity of SWNT than at a greater distance corresponding to the second peak maximum in the distribution. pH lowering strengthens this effect. Since analysis of Figure 11 led to the conclusion that pH lowering resulted in an increase in the distance between CR and DOX while Figure 12 indicated that pH reduction caused an increase in the distance between SWNT and DOX, it means that pH lowering leads to the release of DOX molecule from the structure created by SWNT and CR. Then, free DOX molecules or their small aggregates are present in the solution. Therefore, SWNT-CR system can function as a drug carrier from which the drug is released by a change in pH. 

Figure 13 presents analogical results as Figure 12 for the system containing more CR and DOX molecules. RDF values for CA_SWNT_-CA_CR_ confirm the formation of extensive CR structures at considerable distances from the nanotube and a slightly greater tendency for their creation at low pH. On the other hand, RDF values for CA_SWNT_-CA_DOX_ leaves no doubt as to the fate of DOX molecules earlier (at neutral pH) present in the CT-SWNT structure. It can be seen that at acidic pH, DOX molecules are practically absent within the range of interaction with SWNT. Therefore, it is the next and even stronger confirmation of DOX release from SWNT-CR structure after pH lowering.

**Figure 14:**
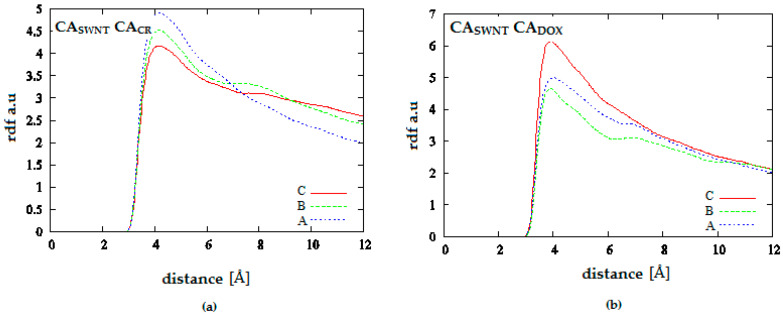
Radial distribution functions for the system SWNT (30.0) complexed with 20 molecules of CR and 10 molecules of DOX; (**a**) SWNT-CR interaction; (**b**) SWNT-DOX interaction; (A—neutral pH, B—medium pH, C—acidic pH).

**Figure 15:**
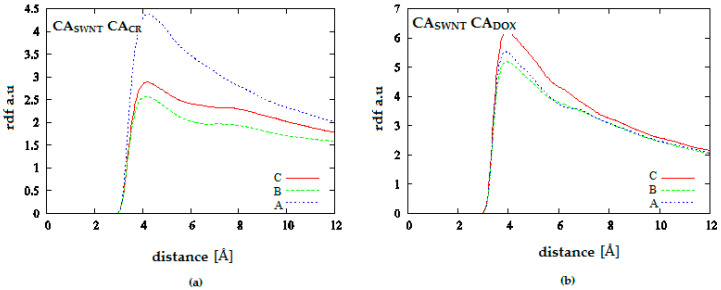
Radial distribution functions for the system SWNT (30.0) complexed with 40 molecules of CR and 10 molecules of DOX; (**a**) SWNT-CR interaction; (**b**) SWNT-DOX interaction; (A—neutral pH, B—medium pH, C—acidic pH).

Analogical analysis performed for radial distribution functions in the systems containing nanotube (30.0) leads to the conclusion that DOX release induced by pH lowering does not occur. As can be seen in Figure 14 and Figure 15, both CR and DOX prefer location near the nanotube surface. pH changes cause only rather moderate quantitative changes in distributions of density of both molecules. 

It can be concluded that DOX release from SWNT-CR structure is determined by the presence of supramolecular ribbon-like CR structures attached to the nanotube surface. However, the existence of such ribbon is not necessarily determined by chirality or nanotube diameter. It appears that a ratio of the number of CR molecules to the nanotube surface area is the main factor. Building of the supramolecular ribbon-like structure begins with tight covering of the nanotube surface by CR molecules because individual adsorption of a molecule on the nanotube surface is accompanied by a very high energetic effect. Thus, most probably wide nanotubes can also function in a similar way as that described for nanotube (10.0) but they require higher CR concentrations. 

Since the mechanism of DOX release does not directly engage the nanotube but it is rather based on processes occurring in the supramolecular ribbon-like structure, the question arises whether nanotubes are required for functioning of CR ribbons as the DOX carrier. The analysis of properties of CR alone in solution allows for the conclusion that the presence of carbon nanotube is indispensable. DOX has to be bound to the CR structures at neutral pH while at this pH CR molecules form small aggregates with insufficient ability to bind DOX. The presence of SWNT stimulates the formation of large structures at neutral pH due to a strong interaction of CR with SWNT thereby increasing the local density of CR molecules which enables the formation of a large supramolecular ribbon-like structure at neutral pH. DOX molecules in the form of small aggregates are incorporated into the ribbon structure at neutral pH since intermolecular CR–CR interactions have a significant repulsive component due to uncompensated charge of deprotonated CR form in solution. When DOX is incorporated into CR structure, these repulsive interactions are reduced to some extent, and thus, the system becomes thermodynamically more stable. The transition of CR from deprotonated to protonated form (pH lowering) eliminates electrostatic repulsive forces and strengthens interactions between CR molecules. This causes the removal of DOX molecules from the supramolecular ribbon-like structure of CR since, in this case, its presence destabilizes the whole system. Finally, only slight interactions between DOX aggregates and compact structure of CR ribbon remain. These interactions are too weak to assure stable adsorption of DOX aggregate on SWNT-CR structure. Thus, DOX molecules are released to solution.

## 4. Conclusions

The results of experiments presented in this article performed with the use of DLS method indicate that gradual pH lowering induces changes in the triple SWNT-CR-DOX complex. The change is related with occurring large aggregates of compounds in the lower pH. Initially, the CR-DOX complex is released and when pH is lowered further, DOX alone is released, as well. Despite the differences in the size of hydrodynamic radius of the observed systems resulting from their different dilution, it is important to observe the tendency of the SWNT-CR-DOX aggregation. We also tried to confirm by the DLS method the release of DOX after lowering the pH. In our study, the essence of the experiments carried out was confirmation of the obtained results of molecular modeling. Earlier experiments [12] also showed the phenomenon of DOX release from the presented system at reduced pH. This indicates that the triple SWNT-CR-DOX complex can be used as a drug carrier system capable of drug release controlled by pH change. The results of molecular modeling calculations for the SWNT-CR-DOX system lead also to the conclusion that the SWNT-CR system can function as a carrier for doxorubicin able to release the drug after pH lowering, and more precisely, after the transition of CR from deprotonated to protonated form. For this to happen, a layer of CR molecules has to be adsorbed onto the nanotube surface and, additionally, a large number of CR molecules has to be present in solution which will form supramolecular ribbon-like spatial structure attached to carbon nanotube. Such a system at a pH corresponding to the deprotonated CR form can incorporate DOX molecules into the ribbon structure. The transition of CR into protonated form considerably stabilizes interactions between CR molecules which results in elimination of DOX molecules from the ribbon structure and its release directly to solution. Calculations proved that such mechanism operates in the case of small-diameter nanotubes (10.0). It is not excluded that a similar mechanism can function for wider nanotubes or multi-wall nanotubes, provided that favorable conditions are created for the formation of a supramolecular ribbon-like structure of CR molecules attached to the nanotube surface. The presence of nanotubes is essential for the efficient binding and release of DOX because supramolecular CR form is more stable when it is attached to SWNT. 

## Figures and Tables

**Table 1:** The average energies of interaction between different components of the SWNT-CR-DOX system. Energies are expressed in kJ mol^−1^ and were calculated for pairs of molecules.

Formulation Energy kJ/mol	SWNT (10.0) + 20CR + 10DOX	SWNT (10.0) + 50CR + 20DOX	SWNT (30.0) + 20CR + 10DOX	SWNT (30.0) + 40CR + 10DOX
Neutral pH				
U_CR-CR_	486	1010	278	451
U_CR-SWNT_	−87	−57	−216	−198
U_DOX-DOX_	−731	−700	−788	−786
U_DOX-SWNT_	−46	−10	−152	−168
U_CR-DOX_	−36	−20	−12	−9
Intermediate pH (between Neutral and Acidic pH)				
U_CR-CR_	−172	-	−236	−241
U_CR-SWNT_	−74	-	−207	−119
U_DOX-DOX_	−748	-	−773	−800
U_DOX-SWNT_	−35	-	−142	−156
U_CR-DOX_	−27	-	−7	−7
		Acidic pH		
U_CR-CR_	−703	−720	−646	−647
U_CR- SWNT_	−48	−51	−201	−141
U_DOX-DOX_	−803	−757	−776	−777
U_DOX-SWNT_	−53	0	−180	−186
U_CR-DOX_	−5	−2	−0.2	-2

## Abbreviations

aiREBO	adaptive intermolecular reactive empirical bond order potential,
AMBER	assisted model building with energy refinement,
CHARMM	chemistry at Harvard macromolecular mechanics,
CR	Congo red,
CR-DOX	Congo red complexed with doxorubicin,
DLS	dynamic light scattering,
DOX	doxorubicin,
GAFF	generalized AMBER force field,
LAMMPS	open source code (Large-scale Atomic/ Molecular Massively Parallel Simulator),
MD	molecular dynamics,
RDF	radial distribution function,
RESP	restrained electrostatic potential,
SEM	scanning electron microscopy,
SRLS	self-assembled ribbon-like structures,
SWNT	single walled carbon nanotubes, carboxylic acid functionalized,
SWNT-CR	single walled carbon nanotubes, carboxylic acid functionalized complexed with Congo red,
SWNT-CR-DOX	single walled carbon nanotubes, carboxylic acid functionalized complexed with Congo red and doxorubicin.
